# Transcranial Sonography in Mitochondrial Membrane Protein-Associated Neurodegeneration

**DOI:** 10.1007/s00062-017-0577-9

**Published:** 2017-03-28

**Authors:** Marta Skowronska, Tomasz Kmiec, Anna Czlonkowska, Iwona Kurkowska-Jastrzębska

**Affiliations:** 1 2nd Department of Neurology, Institute of Psychiatry and Neurology, Sobieskiego 9, 02-957 Warsaw, Poland; 2 Department of Neurology and Epileptology, The Children’s Memorial Health Institute, Warsaw, Poland

**Keywords:** Transcranial sonography, Neurodegeneration with brain iron accumulation (NBIA), Mitochondrial membrane protein-associated neurodegeneration (MPAN), Transcranial sonography of MPAN, MPAN diagnosis, Hyperechogenicity of basal ganglia

## Abstract

**Introduction:**

Although the nature of basal ganglia hyperechogenicity in transcranial sonography (TCS) examinations remains unclear, many studies have shown associations between hyperechogenicity and iron accumulation. The role of iron in basal ganglia hyperechogenicity raises interest in the use of TCS in forms of neurodegeneration with brain iron accumulation (NBIA). Here we analyzed TCS and magnetic resonance imaging (MRI) findings among patients affected by one type of NBIA, mitochondrial membrane protein-associated neurodegeneration (MPAN).

**Methods:**

Investigations using MRI and TCS were performed on 13 patients exhibiting a *C19orf12* gene mutation.

**Results:**

The use of T2/T2* MRI revealed hypointense lesions restricted to the globus pallidus and substantia nigra. Using TCS examination, 12 patients exhibited bilateral hyperechogenicity of the lenticular nucleus, while no patients showed substantia nigra hyperechogenicity.

**Conclusion:**

Investigations with TCS revealed a distinctive hyperechogenicity pattern of the basal ganglia in MPAN patients, which might be useful for differential diagnostics. The variable TCS imaging findings in NBIA patients may result from the presence of different iron content, iron binding partners, such as ferritin and neuromelanin, as well as structural changes, such as gliosis.

## Introduction

Transcranial sonography (TCS) is a simple and safe technique that appears to be useful in evaluating and diagnosing various neurodegenerative disorders. It is employed as an additional tool for diagnosing idiopathic Parkinson’s disease (PD), since substantia nigra (SN) hyperechogenicity is detectable in approximately 90% of patients even in the very early clinical stages of PD [1]. TCS is also presently considered an additional diagnostic tool for corticobasal degeneration (CBD), Wilson’s disease (WD), primary restless legs syndrome (RLS) [2–4] and multiple sclerosis (MS) progression [5]. This technique can detect hyperechogenicity of midbrain structures, including the thalamus, lenticular nucleus (LN), and caudate nucleus, and can be used to determine the diameter of the third ventricle or the frontal horn of the lateral ventricle [2]. The exact nature of basal ganglia hyperechogenicity is unclear, but many post-mortem and animal studies show associations between hyperechogenicity and iron and/or other ion accumulation diseases [6–8]. This potential role of iron in basal ganglia hyperechogenicity raises great interest in the TCS findings in cases of neurodegeneration with brain iron accumulation (NBIA). The NBIAs are a group of genetic conditions characterized by a progressive hypokinetic and/or hyperkinetic movement disorder along with excessive iron deposition in the brain, particularly in the globus pallidus (GP) and SN [9]. The major known forms of NBIA are pantothenate kinase-associated neurodegeneration (PKAN), *PLA2G6*-associated neurodegeneration (PLAN) and now mitochondrial membrane protein-associated neurodegeneration (MPAN). The PKAN is caused by mutation of the pantothenate kinase 2 (*PANK2*) gene and accounts for about half of all NBIAs [10]. The MPAN was recently found, accounts for a substantial number of NBIA cases [11–13], and is caused by a mutation in the orphan gene *C19orf12*, which encodes a mitochondrial protein. The role of C19orf12 remains unknown, but is likely that C19orf12 is involved in the same metabolic pathway implicated in PKAN and PLAN, essential mitochondrial function [14], coenzyme A metabolism and impaired lipid and myelin synthesis [15, 16]. The clinical progression of MPAN is similar to that of classical PKAN, but with a later age of onset and milder symptoms [11, 17, 18]. In the present study, we investigated MRI features and TSC findings among 13 patients with confirmed MPAN.

## Methods

This study included 13 patients with a mutation in the orphan gene *C19orf12.* Of these patients, 10 carried the homozygous deletion c.204_214del11 (Gly69ArgfsX10) in both alleles, and 3 carried the deletion in combination with different missense mutations (*p*.Gly53Arg, and *p*.Thr11Met) in the compound heterozygous state. All included patients gave their written informed consent to participate. Patient medical histories, previous and current therapies, and demographic data were recorded. All patients underwent a neurological examination and MRI and TCS were performed on the same day.

Magnetic resonance imaging was performed using a 1.5-T scanner (Philips Achieva, Eindhoven, the Netherlands). T1-weighted (TR = 596 ms, TE = 15 ms) and T2-weighted (TR = 6783 ms, TE = 140 ms) images were acquired in axial planes with 5‑mm slice thickness. Gradient echo T2* images were obtained as a single-echo sequence (TR = 693 ms, TE = 23 ms, flip angle = 20°).

TCS was performed by a sonographer (MS) who was blinded to the MRI results and specific clinical diagnosis, through the preauricular acoustic bone window, using a 2.5-MHz phased-array transducer (Vivid 7, Milwaukee, WI). The chosen ultrasound parameters were a penetration depth of 14–16 cm, and a dynamic range of 50 dB. Image contrast and brightness were adjusted to obtain the best image. The SN echogenic size measurements were performed automatically on axial TCS scans after manually encircling the outer circumference of the SN echogenic area. For the ultrasound system used SN echogenic areas of ≥0.25 cm^2^ were considered hyperechogenic, those ≤0.20 cm^2^ were considered normal, and intermediate sized areas were considered moderately hyperechogenic. The LN and thalami were visualized in the third ventricular plane. The echogenicity of the LN and thalami were classified as hyperechogenic when their echogenicity was more intense than the surrounding white matter. The area of hyperechogenicity was measured by manually encircling the outer circumference of the hyperechogenic area. The width of the third ventricle was measured on the axial scanning plane.

The control group comprised 18 adult patients without neurological symptoms, including 11 women, and with a mean age of 47 years (range 20–73 years). All control patients had normal MRI results. The control group was not age matched with the patients, but iron accumulation increases in the normally aging brain [18, 19], and we can assume that any changes would be more prominent with time. None showed bilateral LN changes, although four showed unilateral LN changes. None showed changes in the thalami. LN hyperechogenicity was found previously in healthy individuals, but had no prognostic value, and no follow-up studies were conducted [20]. As LN hyperechogenicity was found only in 4 controls and only unilaterally there was no correlation made with the study group.

Statistical analysis was performed to investigate the relationship between age and disease duration and LN hyperechogenicity. A generalized linear model (GLM) [21] was chosen as the basic model to compare LN hyperechogenicity among patients. Since the study design involved recording two measurements for both sides, we also included the factor “side” in the model. An optimal model comprising the Gaussian error and the log-link function was chosen from the GLM family based on the Akaike Information Criterion (AIC), and the deviance was examined to ascertain whether the model was sufficient. The calculations were performed using SAS system rel.13.2, *p* value of <0.05 was considered to indicate statistical significance.

## Results

In this study 13 young adults (2 females, 11 males) of Polish Caucasian origin were investigated, 1 patient (no. 10) was born to related parents and 3 patients (nos. 2, 6, and 11) were brothers who had 2 healthy siblings. There was no family history of affected relatives. All patients were born after a normal pregnancy and were assessed for 9 or 10 points on the Apgar scale.

Table 1 presents the patients’ demographic and clinical data. The mean age of onset was 8.5 years (range 3–15 years) and the time between first symptoms and the present study was 13 years (range 8–23 years). Of the patients 12 had spastic paraparesis of the lower extremities, including 5 who had tetraparesis with the lower limbs being far more affected, 10 patients had dysarthria, and 10 had optic nerve atrophy. Of the 13 included cases, 3 showed parkinsonism and 7 showed dystonia, 3 patients had psychiatric symptoms, 1 of which was diagnosed with schizophrenia and 2 with obsessive-compulsive behavior and all of whom were treated with neuroleptics.

**Table 1:** Demographic and clinical data of 13 MPAN patients

Patient	1	2	3	4	5	6	7	8	9	10	11	12	13
Age (years) at time of presentation	18	18	18	18	18	20	20	21	21	23	26	27	31
Age (years) at symptom onset	7	10	6	8	4	10	3	12	11	9	10	4	15
Sex	M	M	M	M	M	M	M	M	M	F	M	M	F
Family history	–	+	–	–	–	+	–	–	–	–	+	–	–
Consanguinity	–	–	–	–	–	–	–	–	–	+	–	–	–
Mutation	c.[32 C>T]+ [204_214del11], *p*.[Thr11Met]+ [Gly69ArgfsX10]	H	H	H	H	H	H	H	H	H	H	c.[157 G>A]+ [204_214del11], *p*.[Gly53Arg]+ [Gly69ArgfsX10]	c.[32 C>T]+ [204_214del11], *p*.[Thr11Met]+ [Gly69ArgfsX10]
Initial symptoms	Gait	Gait visual	Gait	Gait dysarthria	Gait	Gait	Ataxia, gait	Visual	Gait	Gait	Gait	Gait	Psychiatric
Symptoms at the time of presentation													
Pyramidal signs	–	+	+	+	+	+	+	+	+	+	+	+	+
Dystonia	+	–	–	+	–	+	–	+	–	+	+	+	+
Parkinsonism	–	–	+	–	–	–	–	–	–	+	–	–	+
Dysarthria	–	+	+	+	+	+	+	+	–	+	+	–	+
Optic atrophy	–	+	+	+	+	+	+	+	+	+	+	+	–
Axonal neuropathy	+	+	+	+	+	+	+	+	+	+	+	+	+
Psychiatric	–	–	+	–	–	–	+	–	–	–	–	–	+

*H* homozygous mutation: c.[204_214del11]+[=], *p*.[Gly69ArgfsX10]+[=]. The initial symptoms were: gait impairment with sudden falls; vision acuity; psychiatric signs (obsessive-compulsive disorder); Patients 2, 6 and 11 are brothers

Genetic results from Universität München are given in *square brackets*

**Fig. 1:**
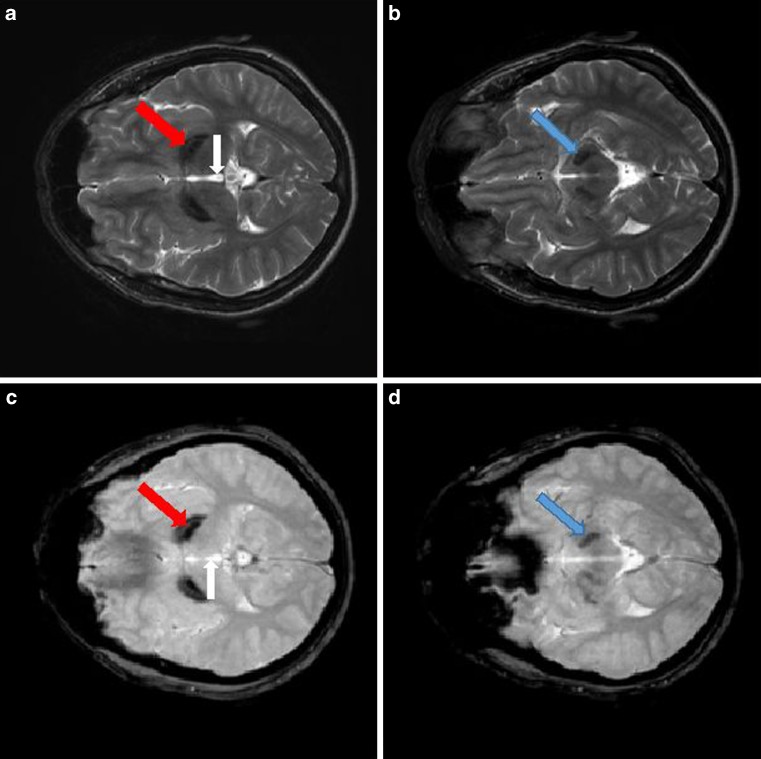
Magnetic resonance imaging results from MPAN patient. **a** and **b** T2-weighted images, **c** and **d** T2*-weighted images. The hypointense signal in globus pallidus (**a** and **c**) and substantia nigra (**b** and **d**) is due to iron accumulation. The third ventricle is indicated by a *vertical arrow*. Notice a medial medullary lamina between the internal and external part of the GP in T2-weighted images – a radiological phenomenon typical for MPAN patients (*red arrow*). Hypointense signal in SN is often less prominent than in GP (*blue arrow*)

In all 13 cases, the T2-weighted and T2* images of the brain showed low signal intensity in the GP and SN bilaterally (Fig. 1). Our results showed no other MRI hypointensity typical of iron accumulation.

**Fig. 2:**
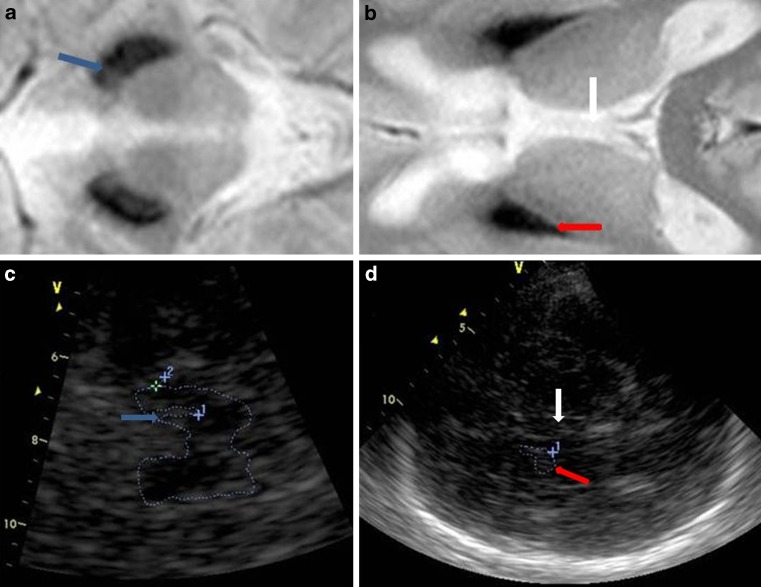
Imaging results from Patient 4. **a** and **b**, T2* MRI images of the substantia nigra (**a**) and globus pallidus (**b**). The third ventricle is indicated by a *vertical arrow*. **c** and **d**, Corresponding transcranial sonography images. The substantia nigra is indicated by an *blue arrow* (**c**). The area of hyperechogenicity is outlined in the medial lenticular nucleus, corresponding to the anatomical globus pallidus (**d**) – *red arrow*

**Fig. 3:**
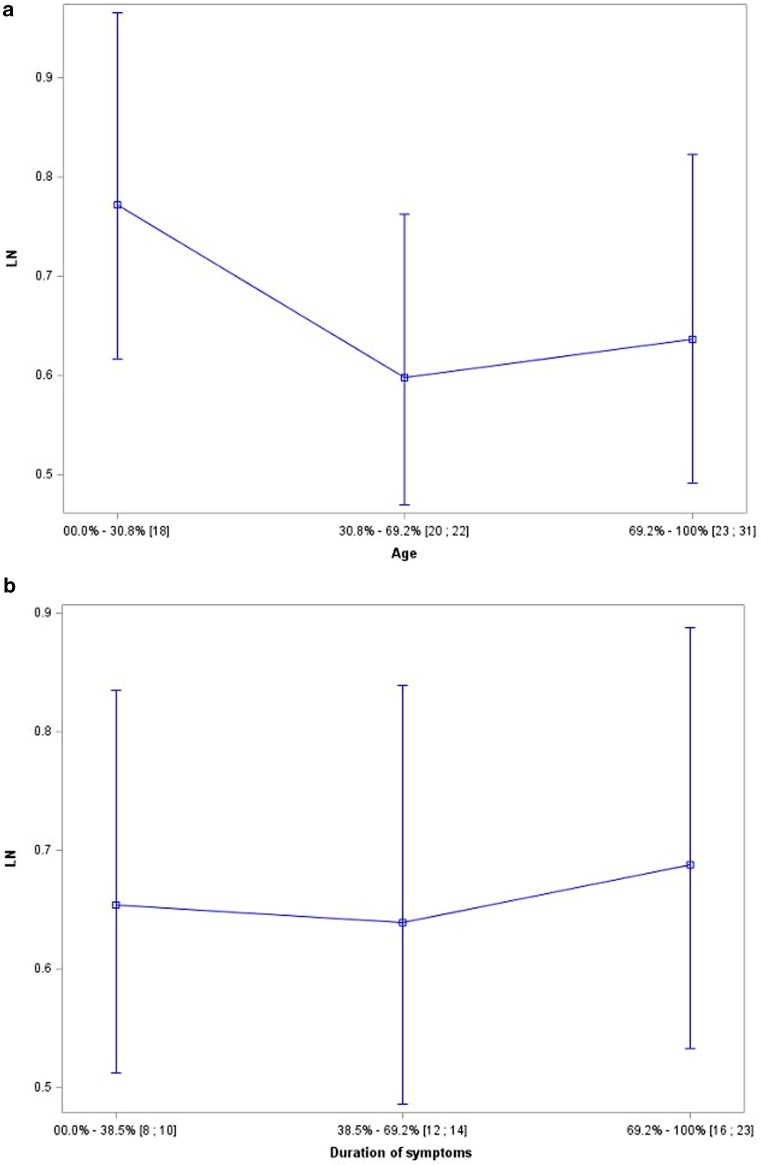
GLM models showing no dependency of LN echogenicity on age (**a**), symptoms duration (**b**). To find the impact of age or symptoms duration patients were divided into 3 groups depending on age (**a**) or symptoms duration (**b**) – the intervals are given in *square brackets*. **a** LN echogenicity decreases when comparing the youngest patients to “middle-age” ones (age 18 years comparing to age 20 to 22 years) and then is stable for the oldest group (23 to 31 years) comparing to “middle-age”, but the changes are not significant. **b** LN echgenicity is stable for first 2 intervals (symptoms duration from 8 to 10 years to symptoms duration from 12 to 14 years) and in the last group with the longest symptoms duration (16 to 23 years) it rises, but the changes are not significant

All patients had a sufficient bone window for TCS. The results of TCS revealed changes restricted to the medial LN, corresponding to the anatomical area of the GP (Table 2; Fig. 2d). Of the patients 12 exhibited symmetrical hyperechogenicity of the LN. Patient number 8 showed no changes in the LN, but had an increased third ventricle diameter. At the follow-up visit after 1 year, TCS examination revealed LN hyperechogenicity in this patient. One case showed moderate hyperechogenicity of the right SN, other patients had normal SN echogenicity (Table 2; Fig. 2c). There was no LN hyperechogenicity area dependency with age, symptom duration (Fig. 3), and clinical phenotype (data not presented).

**Table 2:** Transcranial sonography results in MPAN patients (*n* = 13)

TCS echogenicity (cm^2^)	P.1	P.2	P.3	P.4	P.5	P.6	P.7	P.8 (OCT 2012)	P.8 (DEC 2013)	P.9	P.10	P.11	P.12	P.13
SN (r)	0.19	0.14	0.10	0.11	0.18	0.17	0.13	0.2	0.15	0.12	0.16	0.23	0.16	0.2
SN (l)	0.19	0.11	0.15	0.14	0.12	0.19	0.15	0.2	0.13	0.13	0.17	0.17	0.19	0.2
LN (r)	1.22	1.24	0.80	0.41	0.76	0.48	0.47	–	0.46	0.65	0.37	0.56	0.77	0.9
LN (l)	0.86	0.90	0.82	0.51	0.64	0.61	0.72	–	0.44	0.68	0.28	0.46	0.97	0.7
Thalamus hyperechogenicity	–	–	–	–	–	–	–	–	–	–	–	–	–	–
Third ventricle diameter (mm)	5.8	2.4	2.2	2.7	2.2	1.5	2.5	9.0	9.8	3.1	3.0	2.9	6.2	5.0

*MPAN* mitochondrial membrane protein-associated neurodegeneration, *TCS* transcranial sonography, *SN* substantia nigra, *LN* lenticular nucleus

## Discussion

The NBIA patients have characteristic MRI findings, including hypointense signals on T2 and T2* images due to iron accumulation [9, 18, 22]. Our present MRI findings in MPAN patients showed T2/T2*WI hypointensity in the GP and SN, which is the pattern consistently reported by previous MRI studies in MPAN patients. As in the literature, our MPAN patients showed no involvement of other subcortical structures, such as the putamen, caudate nucleus, thalamus, dentate nucleus, or cortex [11, 12].

No typical pattern has been identified for TCS results in NBIA patients. Our study revealed LN hyperechogenicity corresponding to the GP hypointensity shown in MRI. None of our patients showed SN hyperechogenicity in TCS, despite SN hypointensity on MRIs. We found similar results in a prior study of only three patients [23]. The patient who showed no LN hyperechogenicity in the preliminary study was also patient 8 in our current study, and in this study he showed TCS results typical for MPAN patients. Since the TCS results changed over time in this patient, we speculated that TCS results might be impacted by the duration of the clinical symptoms and/or the patient’s age. Our present analysis showed that LN hyperechogenicity area was not correlated with patient age or symptom duration; however, a limitation of this analysis was that it included only a small group of adult patients, all of whom had experienced symptoms for a few years, and no patients with very recent onset of symptoms. There are no data available of presymptomatic MPAN patients and no conclusions can be drawn when TCS changes can be detected in the disease course. Interestingly, none of our patients showed SN hyperechogenicity in TCS, even though all had obvious changes in MRI. The exact nature of TCS hyperechogenicity in neurodegenerative diseases is not yet understood. Previous studies suggest a correlation with metal accumulation [1–7, 24], which is supported by evidence from post-mortem studies [6, 8]. Among MPAN patients, we would expect TCS hyperechogenicity in both the LN and SN. Kostic et al. performed TCS in patients with PKAN, and found that all patients showed hyperechogenicity in both the SN and LN [25]. On the other hand, Liman et al. examined seven patients with NBIA (including three with confirmed PKAN) and found SN hyperechogenicity in all patients, with no changes in the LN [26]. In an investigation of patients with late-onset NBIA with parkinsonism, Bruggemann et al. found that one patient showed typical MRI findings, along with TCS results of LN hyperechogenicity and normal SN, while genetic tests showed no mutation [27]. Bruggemann et al. also conducted a study within a family carrying a mutation in the *ATP13A2 *gene that is responsible for Kufor-Rakeb syndrome, and showed that the single heterozygous mutation was associated with parkinsonism, but that all mutation carriers showed normal SN echogenicity [28].

In accordance with our present TCS findings, post-mortem analysis of an MPAN patient with a C19orf12 mutation revealed widespread iron deposits in the GP and minimal iron deposition in the SN [10]; however, TCS results vary among different NBIA syndromes despite obvious iron accumulation and similar MRI features regarding GP and SN hypointensity in T2/T2*. Thus, it is possible that hyperechogenicity is influenced not only by metal ions themselves, but also different iron binding partners, such as neuromelanin and ferritin content, which might differ among NBIA patients [7]. Experimental studies in an animal model of PD show that SN hyperechogenicity is caused by structural changes, such as gliosis, rather than by increased iron concentration [29]. Finally, only one of our patients had increased diameter of the third ventricle. This phenomenon is correlated with midbrain structure atrophy and is used for TCS differential diagnosis for progressive supranuclear palsy (PSP) [30]. Brain atrophy is not typical for MPAN patients and was present only in patient no. 8. Together with different LN hyperechogenicity results it suggests slightly different disease course in this case.

The study has some limitations. Firstly, a small study group might be the reason that we failed to show correlations between TCS echogenicity and clinical symptoms. Secondly, the control group was not age matched with patients, but it is unlikely that it influenced the results.

## Conclusion

The use of TCS may be useful for making a differential diagnosis in NBIA patients, but not for primary diagnosis. In particular, TCS can be employed to distinguish between PKAN and MPAN. The variable TCS results among NBIA patients are likely due to different pathomechanisms of iron deposit accumulation/formation and TSC hyperechogenicity is more correlated with metal-binding compounds, inflammation, and demyelination than with metal accumulation.
